# Research on Stray-Light Suppression Method for Large Off-Axis Three-Mirror Anastigmatic Space Camera

**DOI:** 10.3390/s22134772

**Published:** 2022-06-24

**Authors:** Lei Wei, Lin Yang, Yuan-Peng Fan, Shan-Shan Cong, Yan-Song Wang

**Affiliations:** 1 Institute of Frontier and Interdisciplinary Science, Shandong University, Qingdao 266237, China; weilei0906@sdu.edu.cn (L.W.); fanyuanpeng@mail.sdu.edu.cn (Y.-P.F.); wangyansong@sdu.edu.cn (Y.-S.W.); 2 Chang Guang Satellite Technology Co., Ltd., Changchun 130000, China; 15563796171@163.com

**Keywords:** stray-light suppression, scattering model, baffle, off-axis TMA space camera, point source transmittance (PST), veiling glare index (VGI)

## Abstract

The stray-light suppression of a large off-axis three-mirror anastigmatic space camera has been a hot topic, and this study proposes a composite stray-light suppression strategy that effectively suppresses stray light using the combination of a baffle, retaining ring, and internal antistray light measures. Additionally, the light barrier of the third mirror with a three-layered structure was designed to further optimize the composite stray-light suppression system. At the stray-light simulation analysis stage, in view of the limitations of the Torrance–Sparrow scattering analysis model, an analysis model with wide adaptability is proposed, which can be applied to the stray-light simulation analysis of large-size mirrors with rough surfaces. The simulation results indicate that the point source transmittance of the composite stray-light suppression strategy proposed in this paper is of the order of 10^−5^ before installing the light barrier of the third mirror, and the veiling glare index of the full field of view is less than 5.8%. After installing the light barrier of the third mirror, the point source transmittance reached the order of 10^−8^, and the veiling glare index of the full field of view was less than 1.31%. Moreover, the influence of the light barrier of the third mirror on the modulation transfer function of the system was less than 2.3%. The modulation transfer function test of the large-width off-axis three-mirror anastigmatic space camera in a simulated vacuum on-orbit environment was completed, and the test results indicated that the negative impact of the light barrier of the third mirror on the modulation transfer function was less than 3.6%. Moreover, an out-of-field imaging test of the space camera was conducted and the results showed that the image was clear, and the SNR reached 80 dB. The simulation and experimental results prove that the solution in this study can effectively solve the problem of stray-light suppression for large off-axis three-mirror anastigmatic space cameras.

## 1. Introduction

Since the 1980s, off-axis three-mirror anastigmatic (TMA) optical systems have achieved unprecedented development. QuickBird satellite cameras developed by Digital Globe [1,2], EO1 ALI satellite camera developed by NASA [3,4], OSIRIS-NAC camera developed by Astrium of France [5], ALOS satellite PRISM mapping camera developed by Toshiba and Mitsubishi in Japan [6], “tactical optical satellite” camera developed by Surrey and Rutherford Appleton Laboratory (RAL) in the UK [7], Gaia and ProbA-V satellites launched by the European Space Agency [8,9], and a high-resolution hyperspectral imager (HRHSI) proposed by China’s Tiangong-1 adopted an off-axis TMA system as the design scheme. The off-axis TMA space camera has many advantages, such as no chromatic aberration, no center obstruction, a large field of view, and excellent image quality.

The off-axis TMA space camera is developing toward a trend of a long focal length and large aperture. The effective suppression of stray light is a problem that every off-axis TMA space camera must face. Stray-light control and analysis methods for off-axis TMA were systematically described by Clermont and Aballea [10]. With the advancement of additive manufacturing technology, off-axis TMA space cameras will be applied more widely in the future [11]. Stray light can significantly affect the performance of space optical systems, reduce the contrast of the image plane, increase the noise of the system, lead to resolution reduction, and even cause system failure. Presently, most research on the stray-light suppression methods of remote sensing cameras is aimed at small- and medium-sized cameras. The method of combining a baffle with a diaphragm is used to block out-of-field light from directly irradiating the imaging detector, and stray-light paint is sprayed on the inner surface of the baffle to eliminate stray light. The paint method (ERB-2B paint) increased the surface absorptivity and achieved efficient stray-light suppression. However, it is not suitable for large-size off-axis cameras because of the long focal length and large field of view of the camera; the baffle can only be painted and cannot be painted at high temperatures. Additionally, owing to the vibration of the rocket launch stage, the dynamic response of the baffle is high, which leads to the easy peeling off of the spray paint coating, and then contaminates the mirror and imaging detector, making it difficult for large off-axis space cameras to paint the interior of the baffle. Presently, the mirrors of a large-scale space camera are formed by a reaction-sintered silicon carbide (RB-SiC) molding process, which is prone to mirror defects, and it is difficult to ensure good roughness, which further increases the difficulty of suppressing camera stray light. Therefore, it is necessary to study stray-light suppression methods for large off-axis TMA space cameras.

When designing the stray-light suppression system of large off-axis TMA space cameras, it is necessary to model and simulate the designed system. At present, the Harvey–Shack model [12], ABg model, and Torrance–Sparrow model [13] are the most commonly used scattering analysis models, but the three analysis models all have certain limitations. The Harvey–Shack model is only applicable to the analysis of smooth surface analysis, the ABg model is only suitable for modeling and analysis of backscattering on smooth surfaces with nonmechanical structures, and the Torrance–Sparrow model is only suitable for modeling and analysis of small-sized rough surfaces. The above three commonly used simulation models cannot analyze the stray light of large-size off-axis TMA space cameras effectively. Therefore, establishing a new simulation model or perfecting an existing mature simulation model is of great significance to accurately assess the stray-light suppression level of the system.

**Figure 1:**
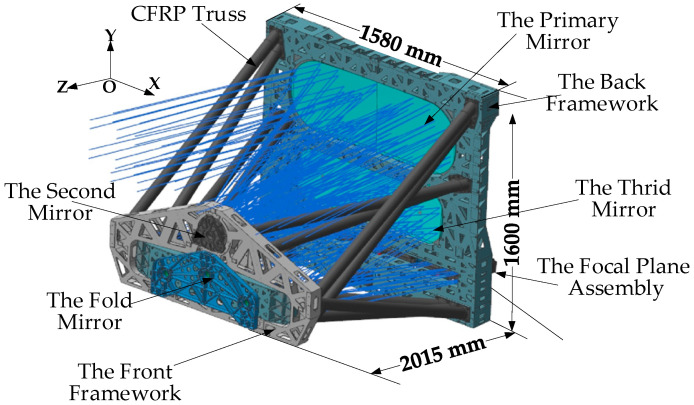
Structure drawing of off-axis three-mirror anastigmatic (TMA) space camera.

The main structure of the space camera studied in this paper is shown in Figure 1, which is mainly composed of the primary mirror, the second mirror, the third mirror, the fold mirror, the front framework, the back framework, the focal plane assembly, and the carbon fiber-reinforced polymer (CFRP) truss. The main parameters of the optical system are shown in Table 1. The aperture of the space camera is 400 mm, the focal length is 4850 mm, the field Angle is 16° × 0.7°, and the spectrum is 450–895 nm. Therefore, the object of this paper is a typical large off-axis TMA space camera. Aiming to address the stray-light analysis of the optical system with a long focal length and large field of view, this study analyzes the factors affecting the stray-light suppression level of the camera, and proposes a composite stray-light suppression strategy, which effectively suppresses the stray light by the combination of the baffle, retaining ring, and internal anti-stray-light measures. On this basis, a light barrier of the third mirror with a three-layered structure is designed to further optimize and iterate the strategy. The limitation of the Torrance–Sparrow stray-light analysis model is analyzed, and a theoretical model with wide adaptability is proposed to analyze the stray light in large-size mirrors with rough surfaces. The stray-light suppression system before and after installing the light barrier of the third baffle is then simulated and analyzed. Meanwhile, the modulation transfer function (MTF) is simulated and analyzed, and the simulation results meet the design requirements. The MTF test and out-of-field imaging experiment of the large off-axis TMA space camera optical system are completed. The results show that the design scheme in this paper can effectively suppress stray light, and the suppression result meets the index requirements.

## 2. Influencing Factors and Evaluation of Stray Light Performance

### 2.1. Introduction to Stray-Light Suppression

According to the source of stray light, it can be divided into external nonimaging stray light, imaging stray light, and internal thermal radiation stray light. External nonimaging stray light refers to the stray light caused by the scattering of external nonimaging light outside the field of view to reach the image plane due to mechanical surface scattering or structural defects in the optical system. Imaging stray light refers to the stray light that is formed when part of the imaging light reaches the image surface through an abnormal optical path inside the optical system due to the residual reflection on the surface of the optical element. Internal thermal radiation stray light mainly exists in the infrared imaging system, where the internal components of the optical system will generate infrared thermal radiation at a certain temperature, which will seriously affect the response of the infrared detector.

The sunlight outside the field of view and the Earth’s reflected light belong to the external nonimaging stray light, and the space camera generally uses the baffle to block. For the scattered light formed by the external stray light irradiating the optomechanical structure and the inside of the baffle, ERB-2B antistray paint (surface sunlight absorption rate of 0.94~0.96) is often sprayed to increase the absorption rate and roughness of the baffle surface and reduce the surface reflectivity of the structure to suppress stray light [14,15,16]. However, the method of spraying antistray paint is only suitable for small- and medium-sized space cameras with good roughness and is not suitable for stray-light suppression in large off-axis TMA space cameras. The main reasons for this are as follows:

(1) The camera was too large to spray paint inside the baffle.

It can be seen from Figure 1 that the size of the optical mechanism structure of the camera is very large, resulting in a very large size of the baffle. At present, the space remote sensing load development unit cannot paint such a huge baffle, but can only use the spray painting process. However, the surface adhesion of spray paint is low, and the spray paint area is large, and is sensitive to vibration. After analysis, under the noise and vibration of the rocket launch section, the random vibration of the local area of the baffle reached 76 Grms, and the spray paint coating has the risk of falling off. Even if the micron-level paint falls off, it will also cause the system’s meter-level resolution to drop. Therefore, for the large off-axis TMA space camera studied in this paper, the outer baffle of the camera will not use the painting process to suppress stray light.

(2) The mirror surface was rough and contained defects.

**Figure 2:**
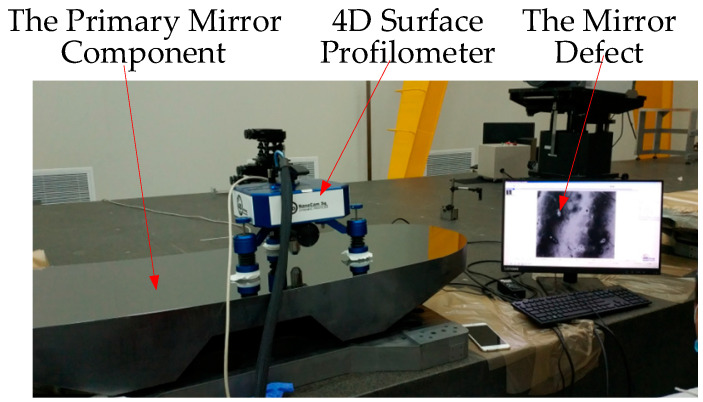
Primary mirror roughness detection site.

The large off-axis TMA space camera studied in this paper has a primary mirror size of 1200 mm × 500 mm. Due to the low specific stiffness of glass ceramics, it cannot meet the lightweight requirements of the mirror, so it can only use the RB-SiC process to develop the mirror blank. The mirror blank developed by the RB-SiC process cannot be directly processed to the optical mirror surface. It needs to be coated with a single crystal silicon layer with a thickness of about 10 μm to improve the surface properties, and then perform the final optical polishing. However, when the RB-SiC process is used for the development of large-size mirror blanks, due to its own process characteristics, if the SiC substrate has large defects, the modified layer will not be able to cover the mirror defects, resulting in poor mirror roughness and small defects after polishing, which makes it difficult to suppress stray light. Figure 2 shows the roughness detection site of the primary mirror of the space camera studied in this paper. The detection results show that the roughness of the mirror studied in this paper can only reach 5 nm, which cannot meet the design requirements of 2 nm, and there are mirror defects, which make stray-light suppression more difficult.

The large size of the camera makes it impractical to spray anti-stray light paint inside the baffle. Meanwhile, the larger roughness of the mirror makes it more difficult to suppress stray light in the large off-axis TMA space camera.

### 2.2. Evaluation System of Stray Light

#### 2.2.1. Point Source Transmittance (PST)

The *PST* is the main indicator for evaluating the stray-light suppression ability of an optical system under different off-axis angles. It is defined as the ratio of the irradiance *E_d_*(*θ*) from the light source, whose off-axis angle is *θ*, to the detector through the optical system and irradiance *E_I_*(*θ*) of the light source at the entrance of the optical system [17].

(1) $$PST=\frac{E_{d}\left(\theta \right)}{E_{I}\left(\theta \right)}$$

The stray-light suppression level of the optical system can be obtained by calculating the *PST* at different off-axis angles in the field of view. The sun can be regarded as a point light source at an infinite distance, and the influence of stray light caused by sunlight is evaluated using the *PST*.

#### 2.2.2. Veiling Glare Index (VGI)

The *VGI* is defined as [18]:

(2) $$VGI=\frac{E_{B}}{E}$$

*E_B_* is the illumination of the stray light on the plane, *E* is the signal light, and *VGI* is the total illumination of the stray light on the image plane. The essence of *VGI* is the proportion of the stray-light energy on the detector to all the light energy reaching the detector, which is a common parameter for describing the stray-light performance of an optical system and can intuitively reflect the size of the stray light of the camera.

Using the two evaluation indicators, *PST* and *VGI,* at the same time, according to their respective characteristics, the size of the stray light of the camera under the condition of point light source and extended light source can be obtained, respectively, which can more comprehensively evaluate the influence of the stray light of the large off-axis TMA space camera studied in this paper.

## 3. Design and Simulation of Composite Stray-Light Suppression System

### 3.1. Design of Composite Stray-Light Suppression System

**Figure 3:**
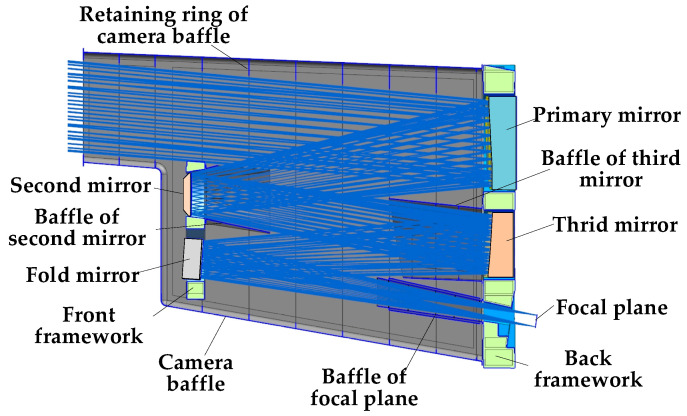
Schematic diagram of the composite stray-light suppression strategy.

Aiming at the large off-axis TMA space camera studied in this paper, a composite stray-light suppression strategy is proposed, which adopts a combination of a baffle, retaining ring, and internal stray-light elimination measures to suppress stray light. A schematic of the composite stray-light suppression strategy is shown in Figure 3. The outer baffle was installed on the rear frame of the space camera, as shown in Figure 1, to prevent stray light outside the field of view from directly shining on the primary mirror, third mirror, and focal plane components. The baffle of the second mirror was installed on the front frame of the camera to block stray light reflected and scattered inside the optical system. The baffle of the third mirror is installed on the rear frame to limit the direct light from outside the field of view to the third mirror, as well as prevent the reflection and scattering of stray light inside the optical system. The baffle of the focal plane was mounted on the rear frame, and the stray light was blocked from the internal reflection and scattering of the optical system to the focal plane detector.

#### 3.1.1. Selection of Baffle Material

The quality requirements of large off-axis TMA space cameras are very strict, and they must resist the harsh vibration environment of rocket launching. Therefore, the material of the baffle should satisfy the characteristics of low weight, high specific stiffness, and high specific strength, simultaneously. To comprehensively compare the performance and process realization of titanium alloy, aluminum alloy, high-volume-fraction SiC/Al, and carbon fiber-reinforced polymer (CFRP) commonly used in space cameras, we chose T700 high-strength CFRP for the development of the baffle. The CFRP used in the baffle designed in this study has a one-way layer thickness of 0.08 mm and the performance parameters are listed in Table 2. The quasi-isotropic laying-mode and laying-angle were [60°/0°/−60°]. Because the baffle is mostly a flat structure, a symmetrical layering method was adopted to prevent warping of the composite laminate. The density of CFRP is 1.78 and the shear modulus is 4.6 GPa. The longitudinal and lateral Poisson’s ratios are 0.27 and 6.55, respectively. The longitudinal and transverse tensile elastic modulus of the material are 132 GPa and 9.5 GPa, respectively.

#### 3.1.2. Structure Design of the Baffle

In theory, a longer outer baffle has the best suppression effect on stray light; however, in practice, the length of the baffle is limited by the envelope of the rocket. Additionally, for large off-axis TMA cameras, GPS antennas, X-band antennas, and other satellite units, they are installed at the upper end of the baffle. If the baffle is too long, the structural stiffness will be small and the vibration response of the single device will be too large. The outer baffle was mainly used to block the reflected light outside the field of view from reaching the light entrance of the camera. The length extending out of the aperture of the hood was [19]:

(3) $$L=\frac{D_{0}}{\tan\gamma -\tan\omega }$$

where *w* is the field of the view of the system and *D*_0_ is the diameter of the light entrance. The light entrance of the off-axis TMA camera was rectangular. According to the camera structure diagram shown in Figure 1, the diameter of the light entrance should be determined in the X-and Y-directions, where γ is the angle of sunlight avoidance.

According to the light direction of the optical system and the main support structure of the camera, the second mirror, focal plane light entrance, and third mirror are provided with inner light baffles to minimize the primary scattered light directly irradiated on the detector.

#### 3.1.3. Design of the Retaining Ring

The retaining ring of the large off-axis TMA space camera has two functions. First, it is used as a stray-light elimination structure to suppress stray light in the system. Second, as a strengthening component of the outer baffle, the stiffness and strength of the baffle are increased, and the vibration response of the outer baffle is reduced to facilitate the loading of components on the satellite.

There are nine, six, and three layers of retaining rings inside the outer baffle, the baffle at the light inlet of the focal plane, and the baffle of the third mirror, respectively. The distance between the retaining ring and edge light was 5 mm, and the height corresponded to the size of the baffles. The 5 mm is obtained according to our engineering experience, and its size is related to machining accuracy, assembling accuracy, and other factors.

#### 3.1.4. Other Stray-Light Suppression Measures

The interior of the baffle of small- and medium-sized space cameras usually needs to be sprayed with anti-stray light paint at an absorption rate of 95% to reduce the stray-light level to the greatest extent. However, for large off-axis TMA space cameras, owing to the large size of the outer baffle, it is impossible to paint; therefore, it can only be dried after natural painting. It has been shown that naturally dried paint layers will fall off when the random vibration response exceeds 45 g. Therefore, the composite stray-light suppression strategy proposed in this paper for large off-axis TMA space cameras will not be used for painting the outer baffle. The stray-light suppression measures and surface properties of other parts of the camera are listed in Table 3. The inner surfaces of the outer baffle, the baffle of the third mirror, and the baffle of the focal plane will be left untouched, leaving the original carbon fiber surface. A suitable amount of ERB-2B paint is sprayed on the inner surface of the second mirror and the inner light entrance of the focusing mechanism to suppress stray light. Other mechanical surfaces will be pasted with heat conductive black film.

### 3.2. Stray-Light Analysis Simulations

#### 3.2.1. Establishment of Torrance–Sparrow Analysis Model with Wide Adaptability

Most of the stray light of the large off-axis TMA space camera is caused by the scattering of the surfaces of the mirrors and mechanical structures. The stray-light suppression level of the system is closely related to the properties of the mirrors and the mechanical structure. The stray-light suppression level of the optical system can be accurately evaluated by modeling the key surface-scattering attributes.

**Figure 4:**
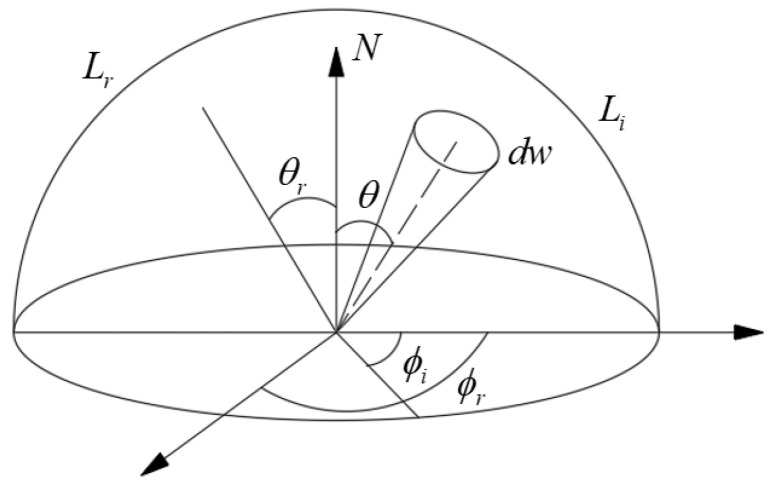
Geometric diagram of bidirectional scattering distribution function.

The scattering attribute of each light is characterized by bidirectional scattering distribution function (*BSDF*) [20] (see Figure 4). The physical meaning of *BSDF* is the ratio of the irradiance of the light emitted from the material surface along a certain direction to the irradiance of the incident light, which can be expressed as [21,22]:

(4) $$BSDF\left(\theta_{i},\phi_{i},\theta_{i},\phi_{r}\right)=\frac{dL_{r}\left(\theta_{i},\phi_{i},\theta_{i},\phi_{r}\right)}{dE\left(\theta_{i},\phi_{i}\right)}=\frac{L_{r}\left(\theta_{r},\phi_{r}\right)}{E_{i}\left(\theta_{i},\phi_{i}\right)}$$

where $\theta_{i}$ and $\phi_{i}$ represent the pitch angle and azimuth angle of the incident light, respectively; $\theta_{r}$ and $\phi_{r}$ represent the pitch angle and azimuth angle of the scattered light, respectively.

The *BSDF* consists of a bidirectional reflectance distribution function (*BRDF*), bidirectional transmittance distribution function (*BTDF*), and bidirectional diffraction distribution function (*BDDF*). The *BRDF* is mainly used to describe the diffuse reflection characteristics of the surface, *BTDF* is used to describe the scattering characteristics of the transparent medium, and *BDDF* is used to describe the scattering characteristics caused by aperture diffraction. For large off-axis TMA space cameras, only the scattered stray light generated by the *BRDF* on the surfaces of the mirrors can be considered in the stray-light analysis, whereas the influence of *BTDF* and *BDDF* on the stray light can be ignored.

When calculating the stray light of an optical system in combination with engineering applications, it is necessary to perform mathematical fitting of the *BSDF* function. Commonly used mathematical models include the Harvey–Shack, ABg, and Torrance–Sparrow models.

The Harvey–Shack model [12] has some limitations. Firstly, the Harvey–Shack model is based on scalar theory and cannot explain the polarization effect of scattered light. Secondly, when the incident angle and scattering angle are large, the analysis results are not accurate. Finally, only when the surface of the medium is smooth, the scattering characteristics are more accurate. The mirror of the large off-axis TMA space camera in this paper adopts the RB-SiC molding process, which is prone to mirror defects and low mirror roughness. Therefore, the Harvey–Shack model is not applicable to the modeling and analysis of scattering characteristics of the large TMA space camera in this paper.

The ABg model is another common scattering light analysis model, but it also has some limitations. Firstly, the ABg model is only applicable to the analysis of the back-scattering characteristics, and the analysis of the forward-scattering characteristics of the surface of optical materials is not obvious, especially when the incident light is irradiated at a large angle. Secondly, the ABg model is not accurate enough to model the surface of mechanical structure materials, which leads to a decrease in the accuracy of the analysis results. Finally, like the Harvey–Shack model, the ABg model is only suitable for smooth surfaces, not for modeling and analysis of the rough material surface. Therefore, it is not applicable to modeling and analysis of scattering characteristics of the large off-axis TMA space camera in this paper.

The Torrance–Sparrow model is a rough surface reflection model based on radiometry and microsurface theory. The microsurface theory believes that a point on the rough surface is composed of many V-shaped concave microsurfaces with different orientations, and countless microplane reflectors are formed with the light incident [13]. The microsurface normal distribution function is expressed as:

(5) $$D\left(h\right)=\exp\left(\frac{{\left(N\cdot H\right)}^{2}-1}{m^{2}{\left(N\cdot H\right)}^{2}}\right)\frac{1}{\pi m^{2}{\left(N\cdot H\right)}^{4}}$$

where *H*, *N*, and *m* are the half-angle vector, the normal vector, and the smoothing factor, respectively.

**Figure 5:**
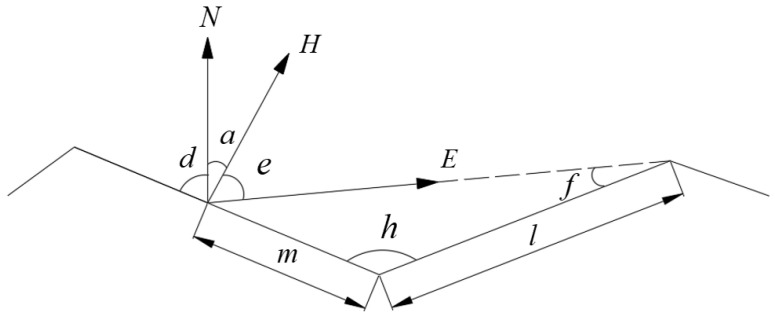
Schematic diagram of the geometry when the light is blocked.

When the Torrance–Sparrow model is used to simulate the microsurface, as shown in Figure 5, it is necessary to model three cases: incident light and outgoing light are not blocked; outgoing light is blocked and incident light is blocked through geometric attenuation factors, and the geometric attenuation factors of the three cases are G, $G_{a}$, and $G_{c}$, respectively.

The BRDF formula of the Torrance–Sparrow model is:

(6) $$BRDF=g\cdot F\cdot \frac{G}{\cos\left(\theta_{i}\right)\cdot \cos\left(\theta_{r}\right)}\cdot \exp\left(-c^{2}\alpha^{2}\right)+\frac{\rho }{\pi }\cos\theta_{i}$$

In the formula, the first term corresponds to specular reflection of small surface elements, and the second term corresponds to diffuse reflection. ρ represents the total reflection ratio, E represents the outgoing light vector, c=E⋅H**,** F represents the Fresnel factor, and g represents the scaling correction factor.

The geometric spreading function can be expressed as:

(7) $$G\left(\theta_{p},\phi_{p}\right)=1-\left(m/l\right)=1-\left[1-{\left(1-A^{2}\right)}^{\frac{1}{2}}\right]/A$$

(8) $$A=\frac{\sin^{2}\phi_{p}-\cos^{2}\left[\left(\phi_{p}-\theta_{p}\right)/2\right]}{\cos^{2}\left[\left(\phi_{p}-\theta_{p}\right)/2\right]-\cos\left[\phi_{p}-\theta_{p}\right]\sin^{2}\phi_{p}}$$

When the Torrance–Sparrow model is used to simulate the microsurface, it is necessary to model three cases: incident light and outgoing light are not blocked, outgoing light is blocked and the incident light is blocked through geometric attenuation factors, and the geometric attenuation factors of the three cases are G, $G_{a}$ and $G_{c}$, respectively.

**Figure 6:**
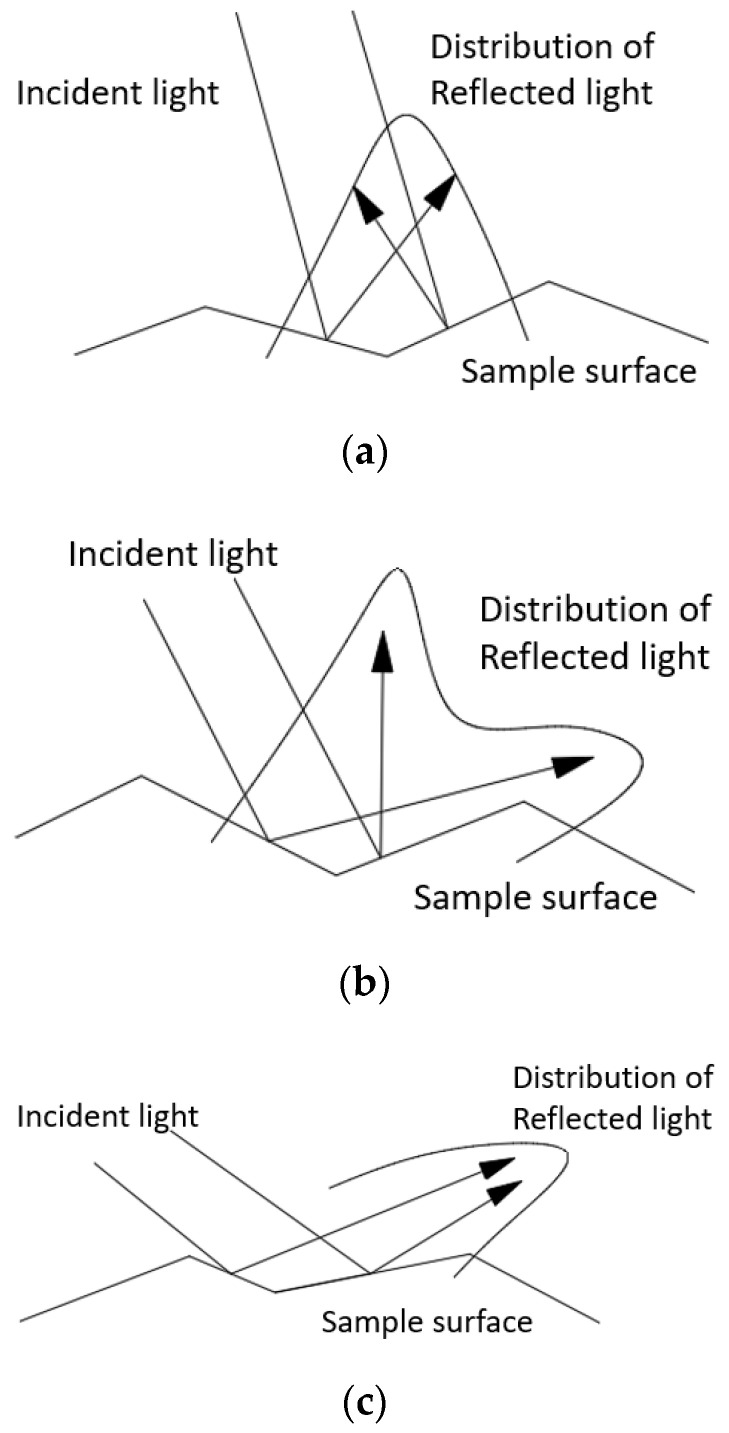
Reflected light distribution under different reflection situations. (**a**) Small angle of incidence; (**b**) medium and large angles of incidence; (**c**) large angle of incidence.

Although the Torrance–Sparrow model is suitable for the modeling and analysis of optical and mechanical systems with high roughness, it assumes that only one reflection is considered for specular reflection. As shown in Figure 6, when a small angle incident occurs, the light is reflected by the two sides of the V-shaped concave in different directions, but the incident angle is small, and the reflection spectrum still shows a peak. When the incident angle increases, the reflected light on the two sides of the V-shaped concave gradually separates, and the reflection spectrum should appear as two peaks, which are nonmirror peaks corresponding to the incident angle of the light source. When the incident angle continues to increase, the two nonmirror peaks gradually approach and tend to combine into one peak.

Therefore, although the traditional Torrance–Sparrow model is suitable for surfaces with high roughness, the accuracy of the stray-light analysis results is reduced when the sample surface size is large and the surface roughness distribution is complex. To accurately model large complex rough surfaces, this study proposes a model with wider adaptability based on the Torrance–Sparrow model.

According to the shadowing effect, nonmirror peaks of medium and large angles are caused by the mirror reflection of the V-shaped concave surface with a slope greater than 1. It should appear at $-\theta_{p}\leq \phi_{p}\leq \phi_{p}^{**}$, where $\theta_{p}$ and $\phi_{p}$ correspond to the incident and reflection angles, respectively. $\phi_{p}^{*}$ and $\phi_{p}^{**}$ correspond to the positive and negative critical angles of the shadowing effect, respectively. In the Torrance–Sparrow model, the nonmirrored peaks produced by the V-shaped concave surface are approximated by a Gaussian distribution. The geometric spreading function is expressed as follows:

(9) $$G\left(\theta_{p},\phi_{p}\right)=b\exp\left\{-a^{2}{\left[\phi_{p}-\beta \left(\theta_{p}\right)\right]}^{2}\right\}$$

where $\beta \left(\theta_{p}\right)$ corresponds to the position of the nonmirror peak, which is a function of the incident angle; a and b are constants.

The geometric spreading function of the wide adaptability Torrance–Sparrow model is presented in Table 4.

The wide-adaptability Torrance–Sparrow model proposed in this study is suitable for the stray-light modeling analysis of large-scale surfaces with poor roughness; thus, it can be applied to stray-light analysis of mirrors in this study. Based on the established Torrance–Sparrow model with wide adaptability, TracePro software was used to analyze the stray light of the large off-axis TMA space camera to determine the VGI and PST.

#### 3.2.2. Simulation Analysis

(1) Stray-light analysis parameter settings

The optical camera is composed of optical (primary mirror, second mirror, third mirror, and fold mirror) and mechanical components. According to the role of the different components in the optical system, the corresponding attributes are assigned to TracePro. The roughness of each reflection surface was set to 5 nm, and the absorption rate of the stray-light elimination structure was set to 0.96. The stray-light analysis properties of the other mirrors are listed in Table 5, and the other structural parts were set to black paint. The reflectance and scattering rate of each component are listed in Table 3.

(2) Point Source Transmittance (PST) and Veiling Glare Index (VGI) analysis.

**Figure 7:**
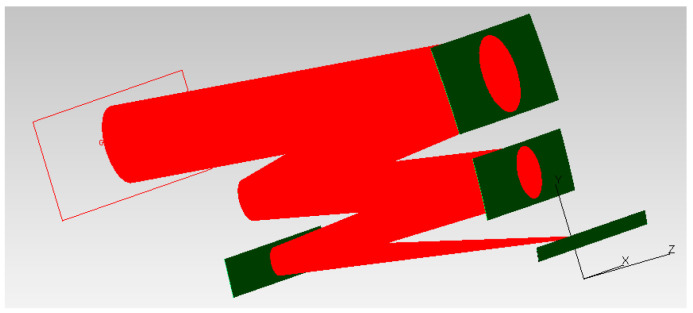
Analysis diagram of TracePro.

In the simulation model, the light source was set as a grid light source located at the exit of the primary mirror baffle, and the light source boundary was a rectangle. The number of tracing rays of the system was 400 million, and the threshold of the trace rays was 10^−8^. By changing the direction of the light beam emitted from the light source surface, the light transmission conditions of different fields of view were simulated. The analysis diagram of TracePro based on the Torrance–Sparrow model is shown in Figure 7.

**Figure 8:**
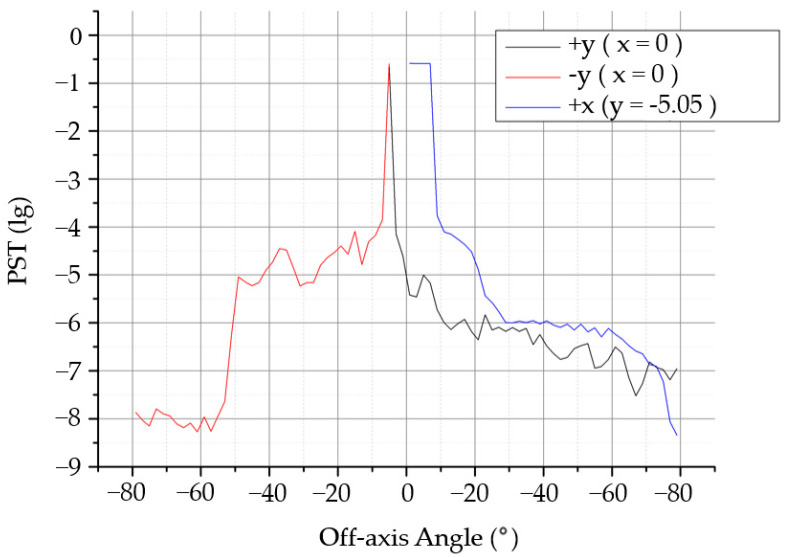
Analysis curve of the modulation transfer function (MTF).

The distribution of the stray light in the image plane outside the field of view was analyzed. When the field of view in the X direction is 0°, the stray light in the Y direction is −5.05~80°, and when the field of view in the Y direction is 5.05°, the stray light in the X direction is 0°–80°. The obtained PST curves are shown in Figure 8. According to the simulation results, when the off-axis angle was 10°–30°, the PST reached an order of 10^−4^, and when the off-axis angle was greater than 30°, the PST reached an order of 10^−5^.

The suppression effect of the composite stray-light suppression strategy was evaluated by calculating the stray-light coefficient. The specific method is to establish a certain size of the area of the light source at different positions of the camera’s focal plane. The area light source was a diffuse light source with an exit half angle of 90°. Using the ray tracing method, a receiving surface covering the entire entrance is set at the entrance of the system to receive the total energy that can reach the image surface. This part of the light includes the effective imaging light and stray light incident on the image plane. Receiving surface 2 is set away from the system, and its field of view is the same as that of the corresponding focal plane small pixel to ensure that the received light is effective in imaging light. The number of rays was set to 4 million and the threshold was 10^−8^. To comprehensively evaluate the stray-light suppression level in the entire field of view, four different positions on the focal plane were selected for the stray light coefficient calculation. The specific positions and parameter settings are listed in Table 6.

The analysis and calculation results of VGI at different field positions are listed in Table 7. In Table 7, the total light energy refers to the total energy obtained by the detector, while the effective light energy refers to the energy remaining except stray light. As shown in Table 7, the VGI of the system is greater than 4%, and the edge field of view is close to 6%, which effectively suppresses the stray light to a certain extent. The calculation results of the VGI prove that the stray-light suppression strategy proposed in this paper is reliable. To further suppress the stray light, based on the wide adaptability Torrance–Sparrow model, the light barrier of the third mirror was designed to further optimize the composite stray-light suppression strategy proposed in this paper.

### 3.3. Further Optimization of the Composite Stray-Light Suppression Strategy

#### 3.3.1. Design of the Light Barrier of the Third Mirror with a Three-Layer Structure

**Figure 9:**
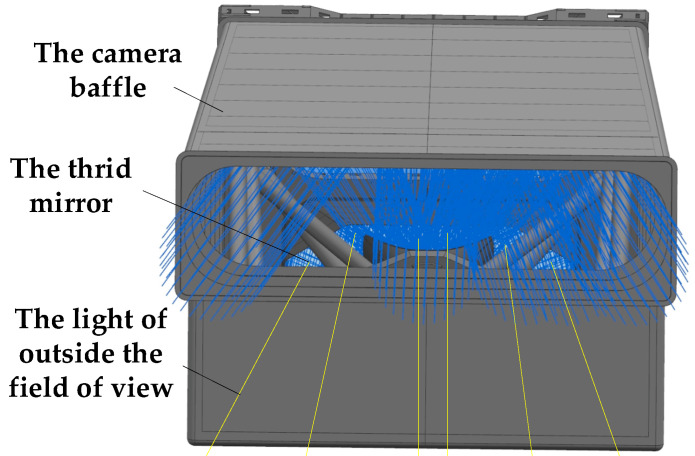
Schematic diagram of the third mirror directly irradiated by the light outside the field of view.

In the large off-axis TMA optical system studied herein, the rockets limit the length of the outer baffle, and the position of the light and the mirror in the optical system limit the structures of the baffle of the third. Therefore, these two factors cause some stray light outside the field of view to directly irradiate the surface of the third mirror without any stray-light suppression, as shown in Figure 9, which causes the stray light to reach the image surface through one or two orders of scattering.

To completely block stray light outside the field of view by irradiating the third mirror, a light barrier with a three-layer structure was designed in this study. The design of the light barrier must meet two principles: first, the influence on the MTF of the system should be less than 5%; second, the structural mass should be small, and structural damage should not occur during the rocket launch stage.

**Figure 10:**
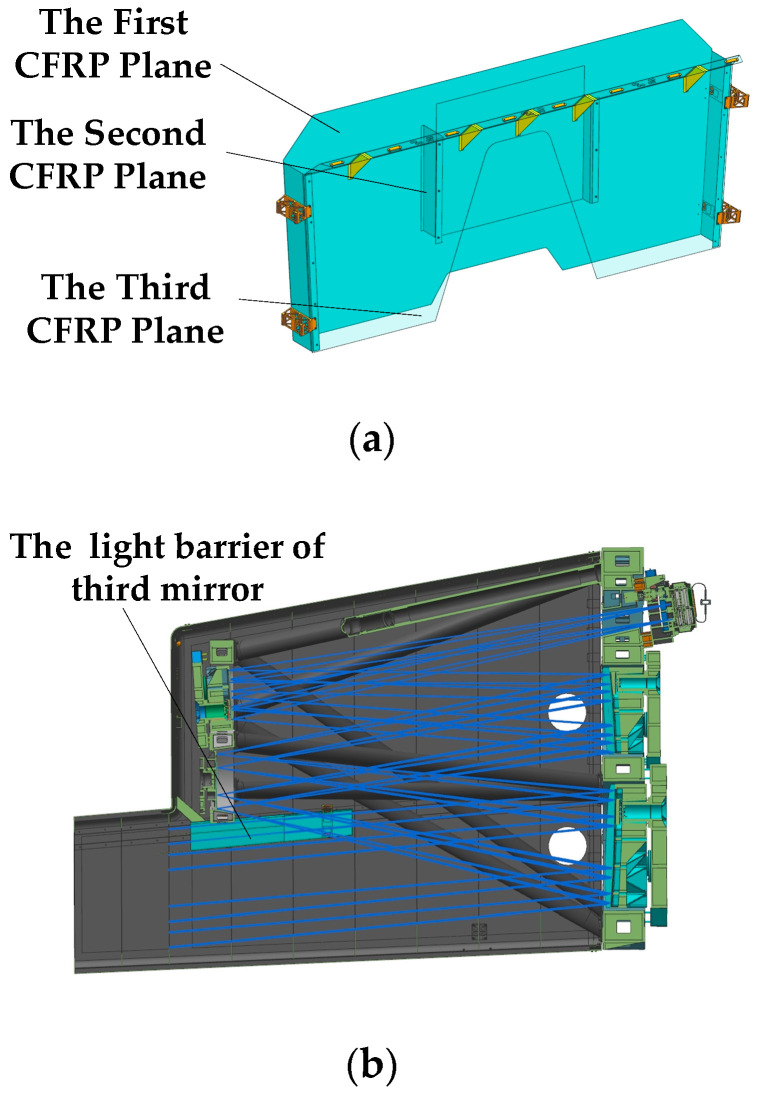

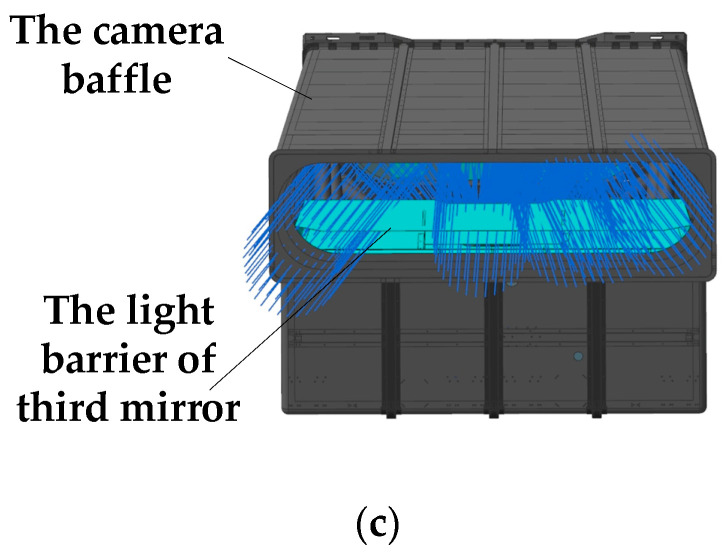
Schematic diagrams of the structure and installation position of the light barrier of the third mirror. (**a**) Structure diagram of the light barrier of the third mirror; (**b**) sectional view of the installation position of the light barrier in the camera; (**c**) schematic of light outside the field of view blocked by the light barrier.

To match the optical system and outer baffle designed in this study, a light barrier with a three-layer structure was designed. The light barrier with a three-layer structure of 1 mm thickness designed in this study is shown in Figure 10a. A schematic of the installation of the light barrier in the optical camera is shown in Figure 10b. As shown in Figure 10c, the stray light outside the field of view in Figure 9 was blocked by the light barrier of the third mirror.

#### 3.3.2. Stray-Light Analysis of the System after the Light Barrier of the Third Mirror Is Installed

**Figure 11:**
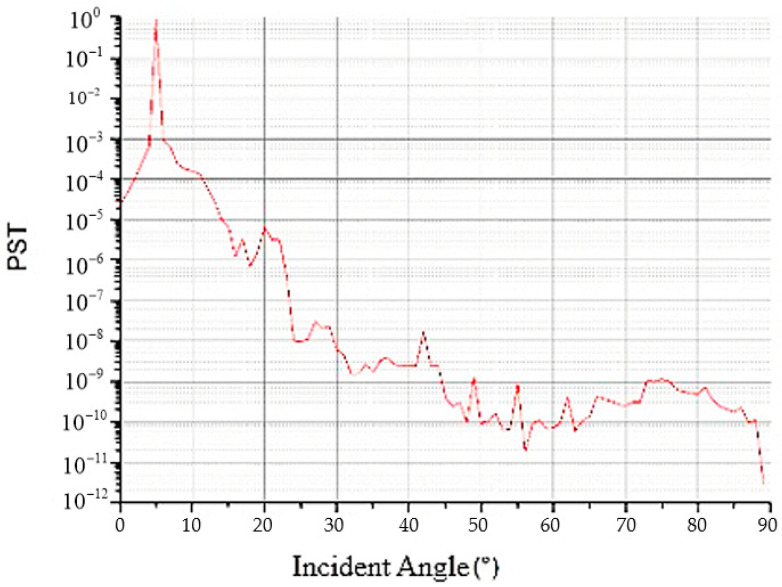
PST analysis curve of the system after installing the light barrier of the third mirror.

A heat conduction black film was pasted on the surface of the light barrier to increase the surface absorption rate and reduce the surface reflectivity. The absorptivity of the barrier was 0.9 and the reflectivity was 0.1. The light-tracing method was used to analyze the stray light of the system after the installation of the light barrier of the third mirror, and the system PST curve shown in Figure 11 and the stray-light coefficient simulation results shown in Table 8 were obtained when the off-axis angle was 30°.

It can be observed from Figure 11 that the illuminance of stray light outside the field of view on the image plane shows a stable downward trend with an increase in the field of view. When the incident angle is greater than 30°, the PST reaches an order of 10^−8^, which is significantly lower than 10^−5^ before installing the light barrier of the third mirror.

As shown in Table 8, the VGI at different field positions is less than 1.31%, which is significantly reduced compared to the installation of the light barrier of the third mirror.

#### 3.3.3. Influence of the Light Barrier on the MTF

**Figure 12:**
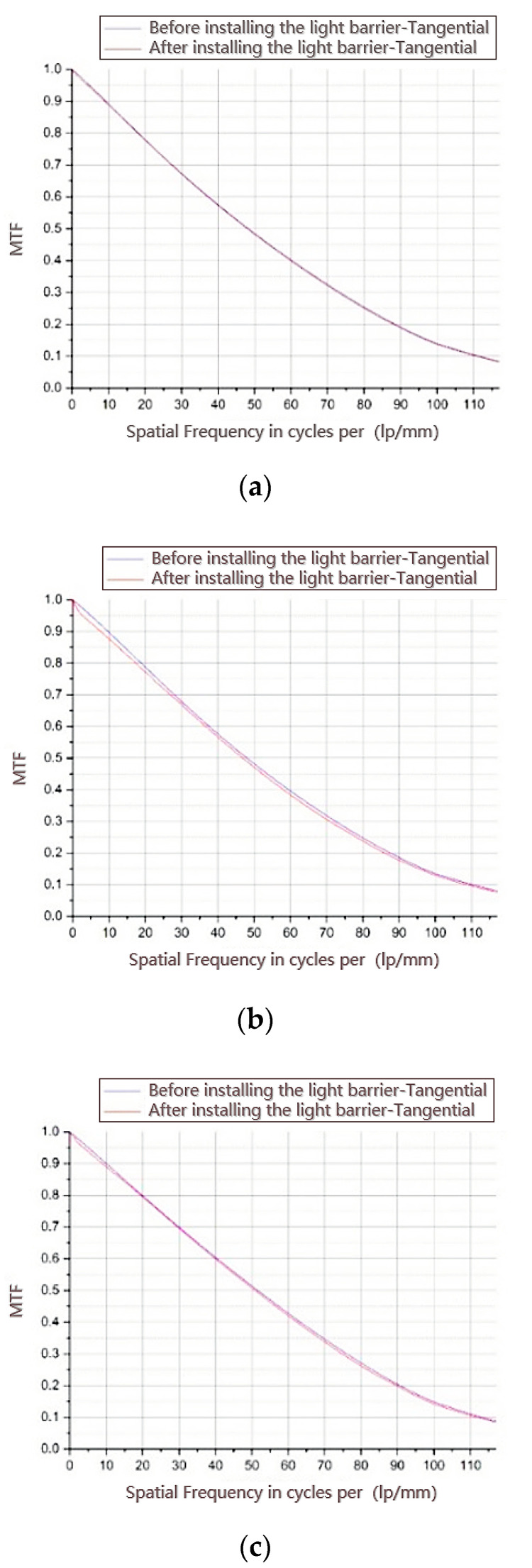

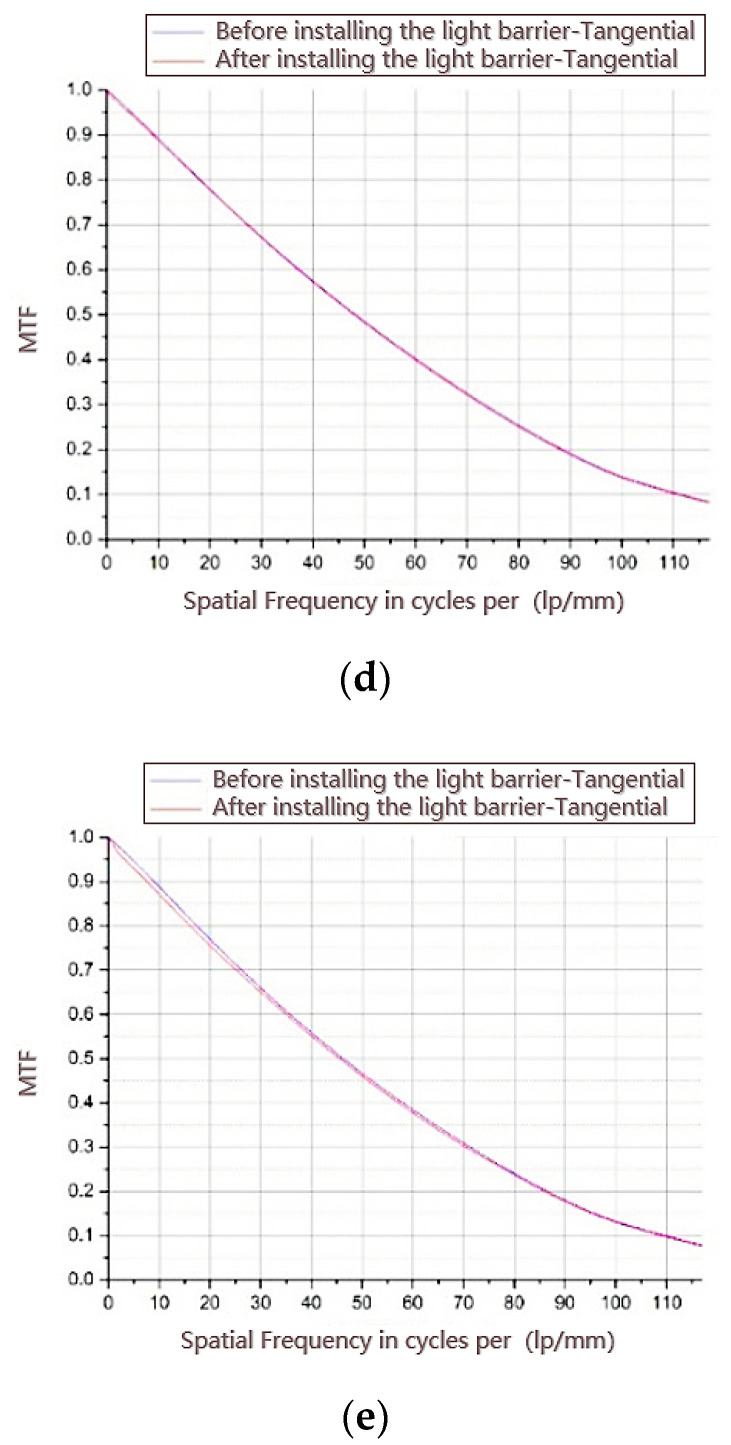
Simulation results of the modulation transfer function (MTF). (**a**) MTF curve in the tangential direction with a field of (0, −5.05°); (**b**) MTF curve in the tangential direction with a field of (0, −4.7°); (**c**) MTF curve in the tangential direction with a field of (0, −5.4°); (**d**) MTF curve in the tangential direction with a field of (4°, −5.05°); (**e**) MTF curve in the tangential direction with a field of (8.05°, −4.7°).

The light barrier of the third mirror blocks the imaging light to a certain extent, resulting in a decrease in the MTF. Because the light barrier has little shading in the sagittal direction, the influence of the light barrier of the third mirror on the MTF in the sagittal direction can be ignored. The influence of the light barrier on the MTF in different fields of view was simulated and analyzed using ZEMAX software, and the results are shown in Figure 12.

It can be observed from the MTF curves in the tangential direction of different fields of view that the light barrier of the third mirror is designed along the central field of view and is thin (1 mm thick); therefore, it will not affect the MTF of the central field of view, but will cause the MTF of the edge field of view to decrease. The maximum decrease in MTF occurs in the field of view of −4.7 ° in the Y-direction edge, which is 2.3% lower than the design MTF at the Nyquist frequency of 50 lp/mm. The specific MTF changes for each field of view are listed in Table 9.

From the above simulation results, it can be observed that the design of the light barrier of the third mirror further strengthens the suppression of the stray light and realizes further optimization of the composite stray-light strategy. The aforementioned stray-light suppression scheme was applied to a large-wide off-axis TMA space camera, and its actual stray-light suppression effect was experimentally verified.

## 4. Experiment

### 4.1. Modulation Transfer Function (MTF) Test

**Figure 13:**
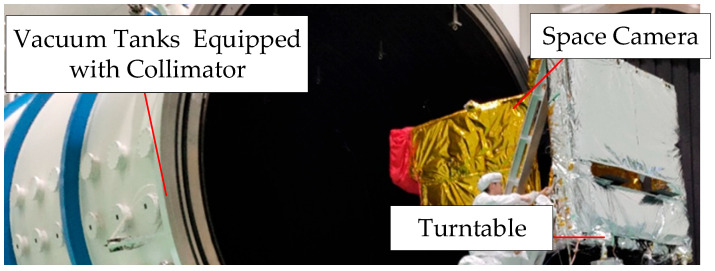
Test site of the modulation transfer function (MTF).

To verify the influence of the stray-light suppression system after installing the light barrier of the third mirror on the MTF of the camera, an on-site MTF test was carried out for the space camera under a simulated vacuum on-orbit environment, and the test site is shown in Figure 13.

**Figure 14:**
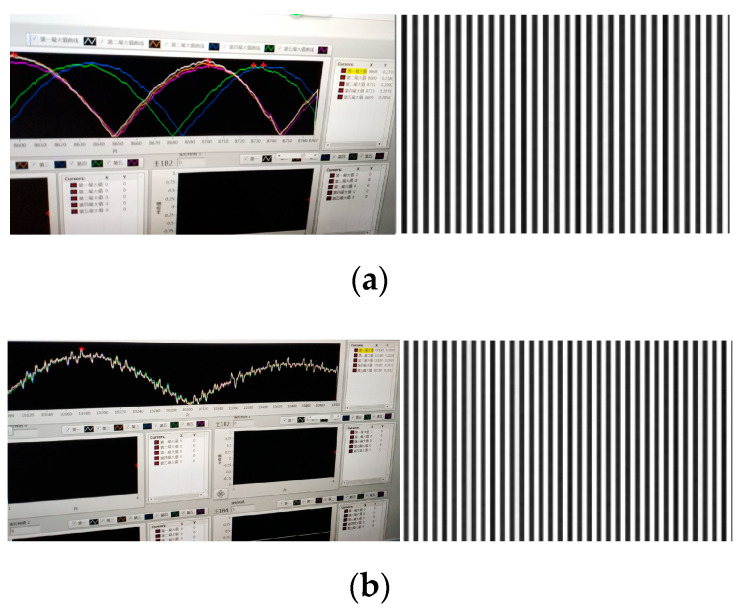
Test curve of MTF and target imaging fringe. (**a**) MTF test results before installing the light barrier of the third mirror; (**b**) MTF test results after installing the light barrier of the third mirror.

The MTF test was performed before and after the installation of the light barrier of the third mirror. The results of the MTF test curve are shown in Figure 14 and Table 10 compares the MTF test data.

As shown in Table 10, at a Nyquist frequency of 50 lp/mm, the influence of the three-mirror light blocker on the MTF is no more than 3.6%, which meets the requirement of less than 5%.

In the simulation results, the MTF decreased by 2.3% at a Nyquist frequency of 50 lp/mm, and the influence of the light barrier on the MTF was no more than 2.3%, which has a certain error compared with the actual test value of 3.6%. There are three main reasons for this: first, errors will be introduced in the process of processing and assembly; second, the vacuum in-orbit environment cannot be completely restored, and the error is caused by environmental noise; and third, the limitations of the Fourier transform, interpolation, and derivation algorithms inevitably introduce some errors.

### 4.2. Imaging Test of the Out-of-Field

**Figure 15:**
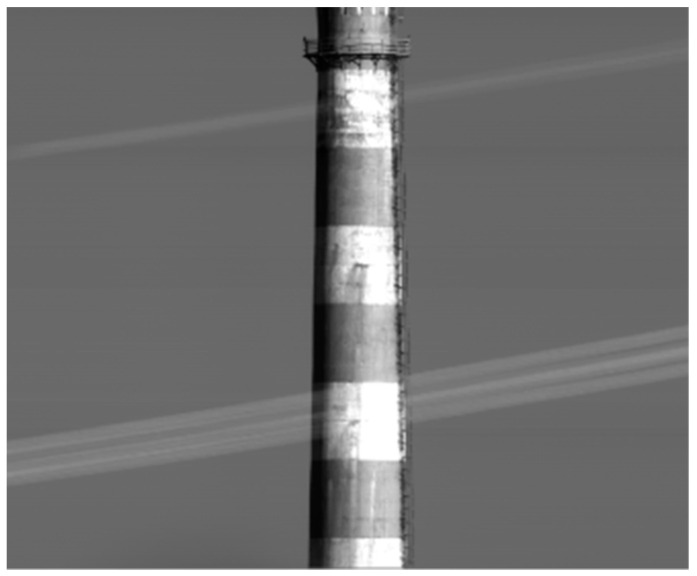
Experimental results of space camera out-of-field imaging.

To verify the effect of the composite stray-light suppression strategy by installing the light barrier of the third mirror on the imaging of the large off-axis TMA space camera, an imaging experiment was performed. The ambient temperature was 15–21 °C. At a distance of 6.4 km, there is a target scene, and the imaging results of the target scene are shown in Figure 15. According to the imaging results in Figure 15, the image is clear with sharp edges and the SNR reaches 80 dB. The experimental results show that the composite stray-light suppression strategy can effectively suppress the stray light of the camera.

## 5. Conclusions

Aiming at the problem of the stray-light suppression ability of the large off-axis three-mirror anastigmatic space camera being insufficient because of its large size and poor surface roughness, a composite stray-light suppression strategy was proposed, which combined a baffle, retaining ring, and internal anti-stray light measures to suppress stray light. In the stray-light simulation analysis stage, in view of the limitations of the Torrance–Sparrow model, an analysis model with wide adaptability is proposed, which can be applied to the stray-light analysis of large optical camera systems with poor roughness. The preliminary simulation analysis of the stray-light suppression system preliminarily designed in this study showed that when the off-axis angle was greater than 30°, the point source transmittance reached the order of 10^−5^, and the veiling glare index of the full field of view was less than 5.8%. To further improve the key parameters, the light barrier of the third mirror with a three-layer structure was designed. After installing the light barrier of the third mirror, the results showed that when the off-axis angle is 30°, the point source transmittance reaches an order of 10^−8^, the veiling glare index of the full field of view is less than 1.31%, and the influence on the modulation transfer function of the system is less than 2.3%. The modulation transfer function test of the large-width off-axis three-mirror anastigmatic space camera in the simulated on-orbit vacuum state is completed, and the results indicate that the influence on the modulation transfer function after installing the light barrier is less than 3.6%, which meets the index requirements. Additionally, the out-of-field imaging test was completed, and the image was clear with sharp edges. The signal-to-noise ratio reached 80 dB, proving that the stray light was effectively suppressed. Based on engineering practice, the problem of stray-light suppression for large off-axis three-reflection space cameras is solved in this paper. The results of an engineering application prove the feasibility and effectiveness of the proposed method. This work provides a reference value for the stray-light suppression of other large-space cameras.

## Figures and Tables

**Table 1:** Parameters of space camera.

Parameters	Numerical Value
Aperture	Φ400 mm
Focal length	4850 mm
Field of view angle of vertical rail working	16° (Width direction)
Field of view angle of the optical system	16° × 0.7° Field of view is 5.05° off center axis
Spectrum	Full spectrum: 450~800 nm
	Blue: 450~510 nm
	Green: 510~580 nm
	Red: 630~690 nm
	Near infrared: 770~895 nm

**Table 2:** Performance parameters of carbon fiber-reinforced polymer (CFRP).

Material	T700 & Epoxy
Density(g/cm³)	1.78
Longitudinal tensile elastic modulus/Gpa	132
Lateral tensile elastic modulus/Gpa	9.2
Longitudinal Poisson’s ratio	0.27
Lateral Poisson’s ratio	6.55
Shear Modulus/Gpa	4.6

**Table 3:** Stray-light suppression measures and surface properties on the inner surface of the large off-axis TMA space camera.

Projects	Treatment Measures	Surface Absorptivity	Specular Reflectance	Integrated Scattering Rate
-Inner surface of the outer baffle	Carbon fiber surface, no treatment	0.9	0.1	0
-Inner and outer surface of the second mirror baffle	Spray ERB-2B paint	0.956	0	0.044
-Inner and outer surface of the baffle of the focal plane entrance	Carbon fiber surface, no treatment	0.9	0.1	0
-Structural surface of carbon fiber truss	Paste heat conductive black film	0.9564	0.002	0.088
-Outer surface of the front frame	Paste heat conductive black film	0.9564	0.002	0.088
-Outer surface of the rear frame	Paste heat conductive black film	0.9564	0.002	0.088
-Inner light entrance of the focusing mechanism	Spray ERB-2B paint	0.956	0	0.044

**Table 4:** Geometric spreading function expression for the wide adaptability Torrance–Sparrow model.

Incident Angle	Reflection Angle	Geometric Spreading Function
$0\leq \theta_{p}\leq \frac{\pi }{4}$	$-\frac{\pi }{2}\leq \phi_{p}\leq \phi_{p}^{**}$	$G\left(\theta_{p},\phi_{p}\right)=1-\frac{m}{l}=1-\left[1-{\left(1-A^{2}\right)}^{\frac{1}{2}}\right]/A$
	$\phi_{p}^{**}\leq \phi_{p}\leq \phi_{p}^{*}$	$G\left(\theta_{p},\phi_{p}\right)=1$
	$\phi_{p}^{*}\leq \phi_{p}\leq \frac{\pi }{2}$	$G\left(\theta_{p},\phi_{p}\right)=1-\frac{m}{l}=1-\left[1-{\left(1-A^{2}\right)}^{\frac{1}{2}}\right]/A$
$\frac{\pi }{4}\leq \theta_{p}\leq \frac{\pi }{2}$	$-\frac{\pi }{2}\leq \phi_{p}\leq -\theta_{p}$	$G\left(\theta_{p},\phi_{p}\right)=1-\frac{m}{l}=1-\left[1-{\left(1-A^{2}\right)}^{\frac{1}{2}}\right]/A$
	$-\theta_{p}\leq \phi_{p}\leq \phi_{p}^{**}$	$G\left(\theta_{p},\theta_{p}\right)=b\exp\left\{-a^{2}{\left[\phi_{p}-\beta \left(\theta_{p}\right)\right]}^{2}\right\}$
	$\phi_{p}^{**}\leq \phi_{p}\leq \phi_{p}^{*}$	$G\left(\theta_{p},\phi_{p}\right)=1$
	$\phi_{p}^{*}\leq \phi_{p}\leq \frac{\pi }{2}$	$G\left(\theta_{p},\phi_{p}\right)=1-\frac{m}{l}=1-\left[1-{\left(1-A^{2}\right)}^{\frac{1}{2}}\right]/A$

**Table 5:** Property settings of optical components.

Surface Absorptivity	Specular Reflectance	Integral Scattering Rate
0.0782454	0.9147846	0.00697

**Table 6:** Settings of light sources in different field of view directions.

Position Selected on the Focal Plane	Correspond to the X-Direction Field of View	Correspond to the Y-Direction Field of View	Size of Small Bin on the Focal Plane (Diameter in mm)
The center of the focal plane	−0.1°~0.1°	−5.15°~−4.95°	17.2
The leftmost part of the focal plane	−8.0°~−7.8°	−5.15°~−4.95°	
The top of the focal plane	−0.1°~0.1°	−4.9°~−4.7°	
The left half of the focal plane	−4.125°~−3.925°	−5.15°~−4.95°	

**Table 7:** Simulation results of the VGI at different small surface light sources.

Area Light Source Position	Total Light Energy/W	Effective Light Energy/W	The VGI
The center of the focal plane	0.00060655	0.00058047	4.3%
The leftmost part of the focal plane	0.0003518	0.00033139	5.8%
The top of the focal plane	0.00059145	0.00056661	4.2%
The left half of the focal plane	0.00062491	0.00059429	4.9%

**Table 8:** Simulation results of the VGI after installing the light barrier of the third mirror.

Area Light Source Position	Total Light Energy/W	Effective Light Energy/W	The VGI
The center of the focal plane	0.00060655	0.0006	1.08%
The leftmost part of the focal plane	0.0003518	0.00034718	1.31%
The top of the focal plane	0.00059145	0.00058612	0.90%
The left half of the focal plane	0.00062491	0.00061783	1.13%

**Table 9:** Statistical table of MTF changes in different fields of view in the tangential direction.

Field of View/(°)	MTF Value before Installing the Light Barrier	MTF Value after Installing the Light Barrier	Change of MTF
0, −5.4	0.513943	0.507368	0.006575
4, −5.4	0.484876	0.481725	0.003151
8.05, −5.4	0.391528	0.386436	0.005092
0, −4.7	0.481841	0.470725	0.011116
4, −4.7	0.500093	0.490409	0.009684
8.05, −4.7	0.466941	0.460235	0.006706
0, −5.05	0.504845	0.504845	0
4, −5.05	0.492637	0.491807	0.00083
8.05, −5.05	0.438936	0.438936	0

**Table 10:** MTF test results of Panchromatic band.

Position	Before Installing the Light Barrier	After Installing the Light Barrier	Influence on MTF/%
Focal plane center	0.213	0.214	0.47
Left side of focal plane	0.207	0.203	1.9
Right side of focal plane	0.194	0.186	3.6

## Data Availability

Not applicable.
